# Supplementary material for: The MAL Family of Proteins: Normal Function, Expression in Cancer, and Potential Use as Cancer Biomarkers
Source: Cancers (Basel). 2023 May 17;15(10):2801. doi: 10.3390/cancers15102801 (PMC10216460; doi:10.3390/cancers15102801)
Supplement: Supplementary file 1 [file cancers-15-02801-s001.zip › cancers-2279285-supplementary.pdf]

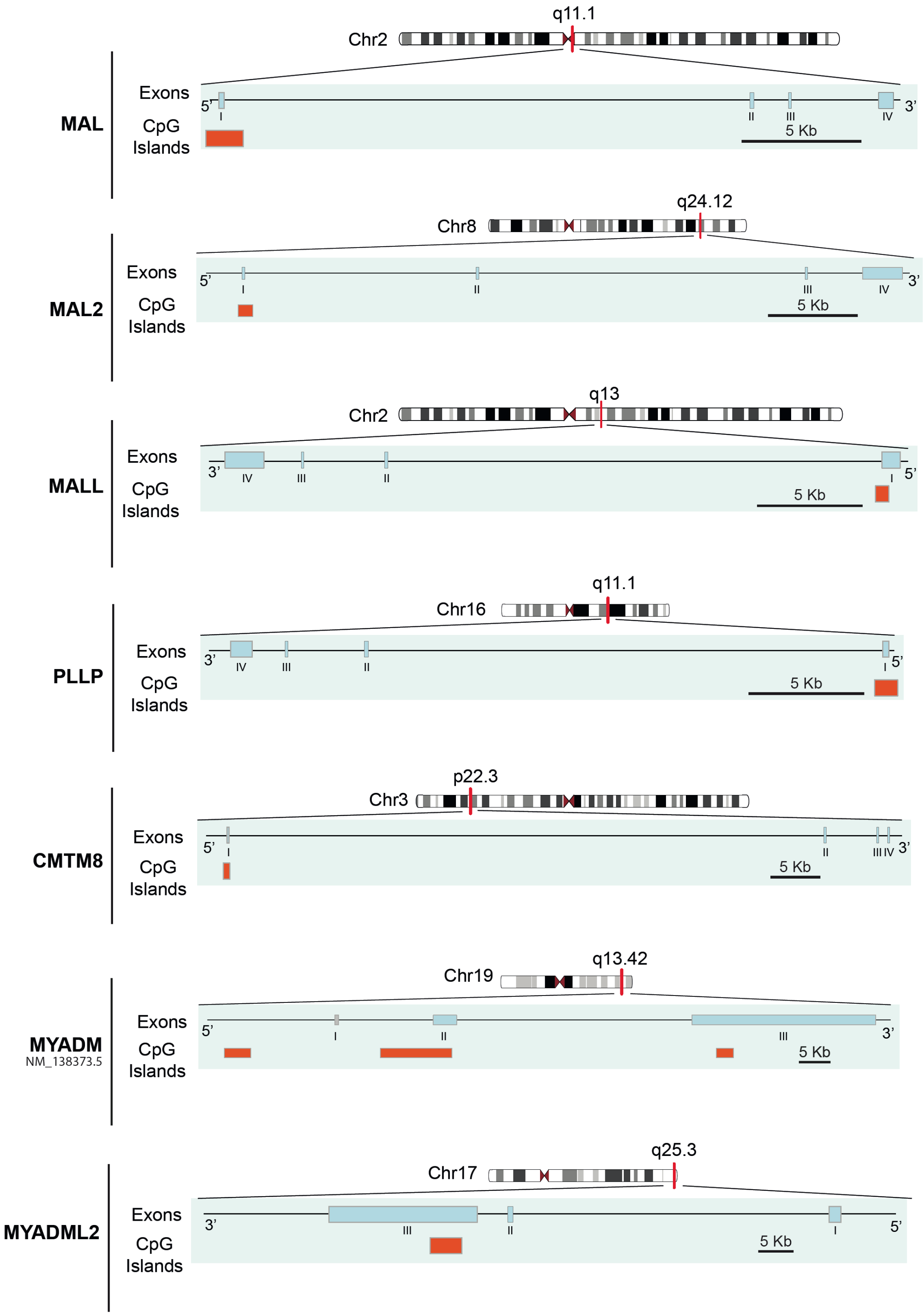

Figure S1. Chromosome, gene structure, and CpG islands of MAL-family genes. The chromosome position of each *MAL*-family gene, their exon-intron structure and the existence of CpG islands are indicated. CpG island information was extracted from UCSC Genome Browser on Human (hg19) using R packages.

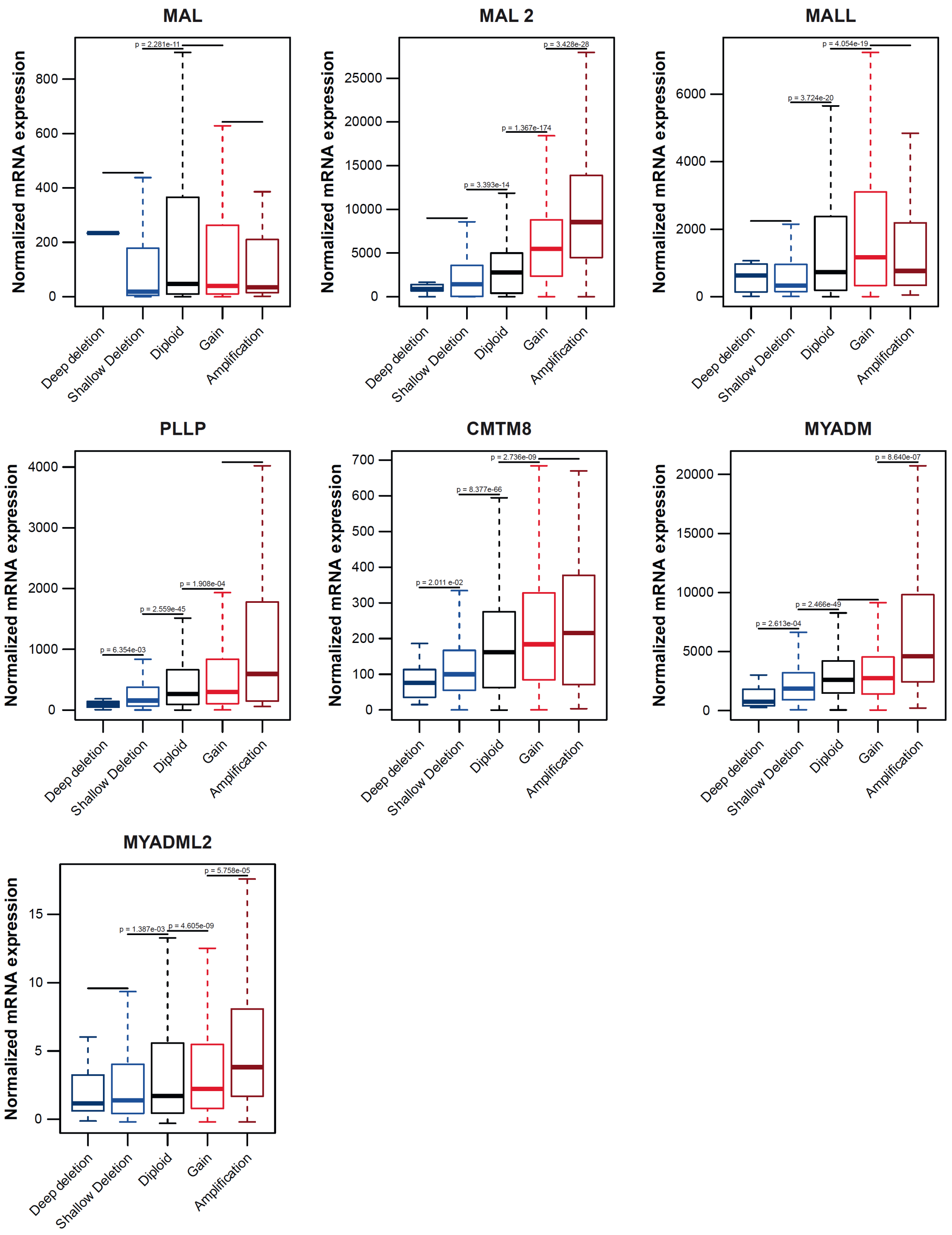

Figure S2. Correlation between *MAL*-family gene expression and copy number alterations. Plots were generated using data downloaded from cBioportal. Deep deletion indicates a possibly homozygous deletion; shallow deletion, a possibly heterozygous deletion; gain, a few additional copies; and amplification, a large number of copies.

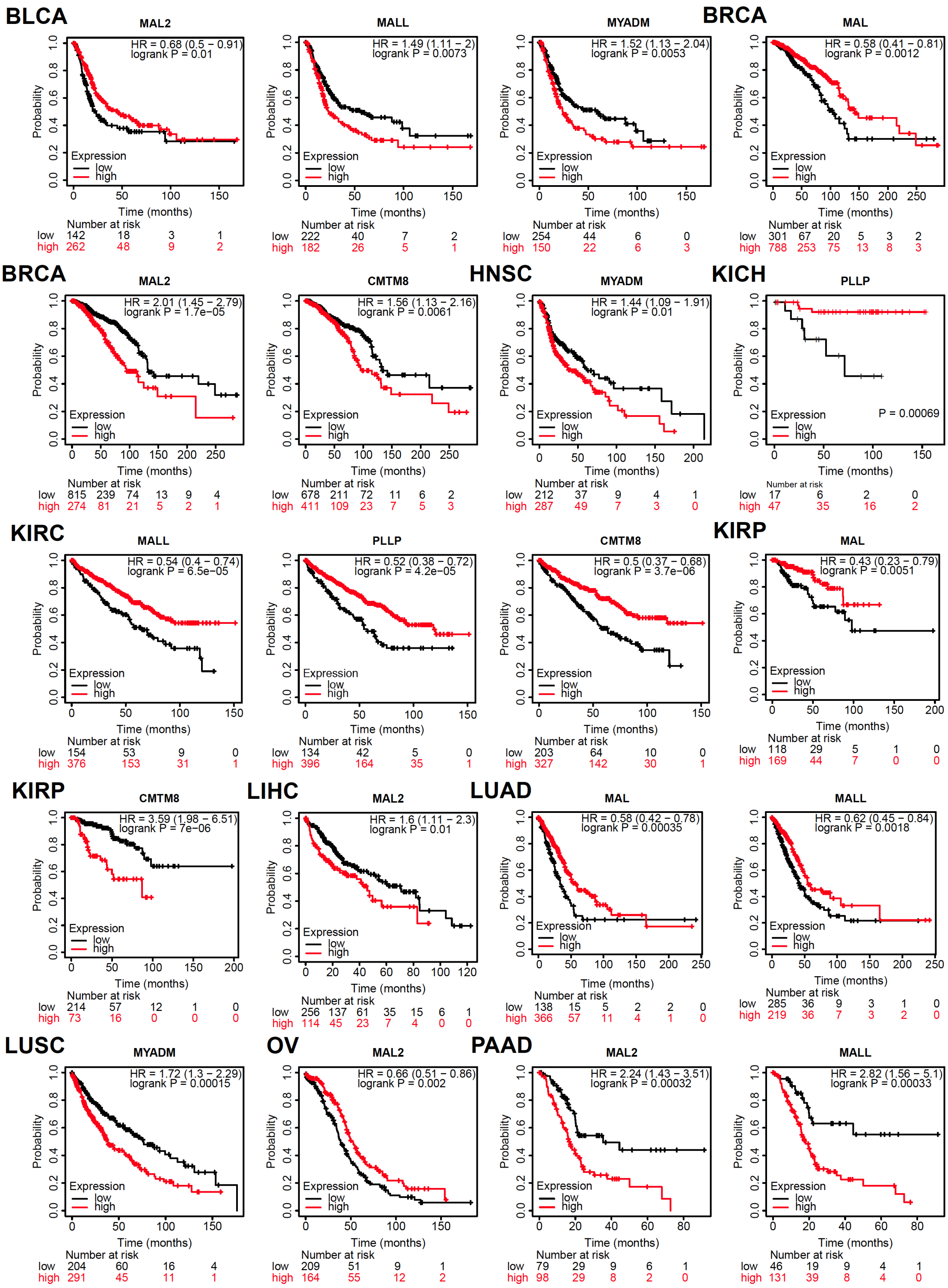
BLCA
BRCA
HNSC
KICH
KIRC
KIRP
LIHC
LUAD
LUSC
OV
PAAD
MAL2
MALL
MYADM
MAL
CMTM8
PLLP
Probability
Time (months)
Number at risk
Expression
low
high

(Cont.)

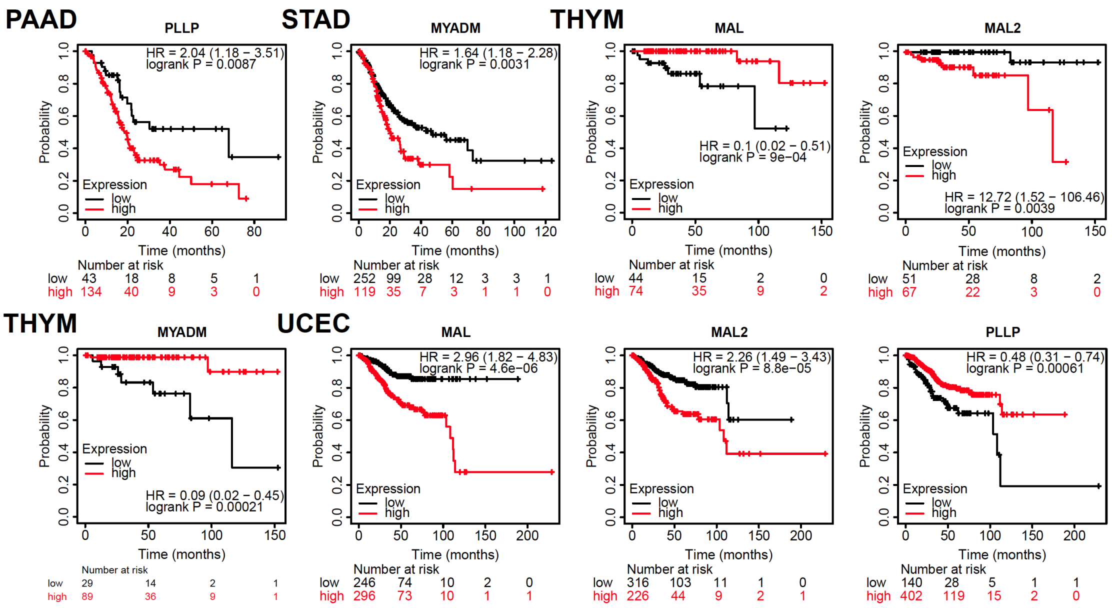

Figure S3. Kaplan-Meier plots showing the correlation between the expression levels of *MAL*-family genes and overall survival. Plots that yielded a log-rank P ≤0.01 are shown. Plots were produced with Kaplan-Meier plotter and pan-cancer datasets, except in the case of KICH, which was obtained with HPA software using TCGA data. The best expression cut-off was used to determine the maximum difference with regard to survival between the groups of high and low levels of gene expression for the lowest log-rank P value. HR, hazard rate. BLCA, bladder urothelial carcinoma; BRCA, breast invasive carcinoma; HNSC, head and neck squamous cell carcinoma; KICH, kidney chromophobe carcinoma; KIRC, kidney renal clear cell carcinoma; KIRP, kidney renal papillary cell carcinoma; LICH, liver hepatocellular carcinoma; LUAD, lung adenocarcinoma; LUSC, lung squamous cell carcinoma; OV, ovarian serous cystadenocarcinoma; PAAD, pancreatic adenocarcinoma; STAD, stomach adenocarcinoma; THYM, thymoma; UCEC, uterine corpus endometrial carcinoma.

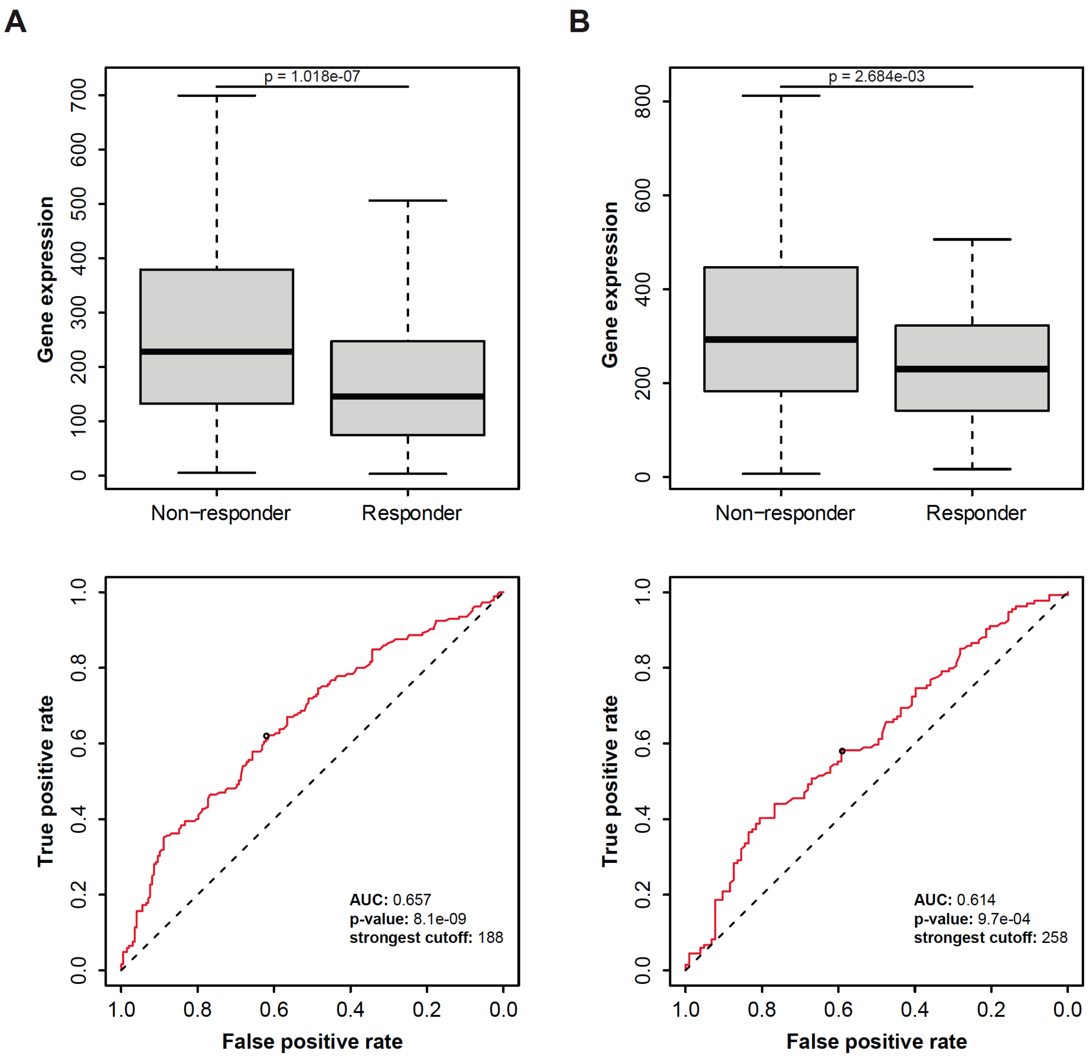

Figure S4. ROC plots of the correlation between *MAL* expression and relapse-free survival at 5 years in breast cancer patients treated with adjuvant chemotherapy. Normalized *MAL* gene expression of non-responders and responders (top panels), and true positive rate (sensitivity) versus false positive rate (specificity) plots (bottom panels) of breast cancer patients treated with anthracycline (A) and taxane (B). AUC, area under the curve. (In A: n = 1368 patients, 933 non-responders and 435 responders; in B: n = 1109 patients, 778 non-responders and 331 responders).

Table S1: Expression levels in TPM of *MAL*-family genes in human cancer

	MAL			MAL2			MALL			PLLP			CMTM8			MYADM			MYADML2		
	Tumor	Normal	Tumor vs Normal	Tumor	Normal	Tumor vs Normal	Tumor	Normal	Tumor vs Normal	Tumor	Normal	Tumor vs Normal	Tumor	Normal	Tumor vs Normal	Tumor	Normal	Tumor vs Normal	Tumor	Normal	Tumor vs Normal
**BLCA**	5.89	11.66	0.51	93.15	32.28	2.89	19.56	31.97	0.61	9.13	9.76	0.94	6.63	4.23	1.57	19.57	107.68	0.18	0.02	0.01	2.00
**BRCA**	0.71	1.84	0.39	124.19	17.34	7.16	6.85	12.82	0.53	4.86	9.66	0.50	11.92	13.76	0.87	84.5	91.65	0.92	0.06	0.04	1.50
**CESC**	3.08	14.83	0.21	135.93	0.28	485.46	66.9	7.46	8.97	12.56	5.14	2.44	4.23	3.36	1.26	20.02	122.38	0.16	0.01	0.02	0.50
**COAD**	0.31	19.88	0.02	121.73	4.24	28.71	39.64	12.45	3.18	6.5	3.76	1.73	15.76	3.14	5.02	49.83	92.28	0.54	0.85	0.04	21.25
**ESCA**	1.42	1653.55	0.00	172.72	184.55	0.94	64.17	242.47	0.26	14.9	24.09	0.62	11.69	2.87	4.07	91.4	16.75	5.46	0.09	0.05	1.80
**HNSC**	4.41	1043.66	0.00	125.88	158.88	0.79	74.43	127.81	0.58	4.79	11.28	0.42	3.11	3.28	0.95	38.15	30.63	1.25	0.04	0.42	0.10
**KICH**	431.98	446.18	0.97	43.7	59.09	0.74	2.67	4.13	0.65	140.84	43.16	3.26	4.24	6.01	0.71	11.99	23.72	0.51	0.01	0.02	0.50
**KIRC**	17.49	398.4	0.04	13.05	81.45	0.16	25.08	5.74	4.37	17.99	38.77	0.46	4.71	5.92	0.80	64.45	29.01	2.22	0	0.01	0.00
**KIRP**	233.11	409.01	0.57	73.87	51.66	1.43	7.08	5.34	1.33	41.03	41.8	0.98	4.95	6.74	0.73	31.54	27.93	1.13	0.01	0.02	0.50
**LIHC**	0.25	0.45	0.56	21.34	11.28	1.89	2.63	1.04	2.53	4.59	3.51	1.31	22.32	15.86	1.41	16.2	9.07	1.79	0.01	0	
**LUAD**	4.48	22.58	0.20	173.49	48.06	3.61	108.99	40.78	2.67	16.83	58.2	0.29	17.17	19.24	0.89	48.82	101.98	0.48	0.04	0.03	1.33
**LUSC**	2.47	22.7	0.11	102.84	46.37	2.22	34.85	40.09	0.87	10.32	55.68	0.19	8.54	18.99	0.45	34.46	102.04	0.34	0.04	0.03	1.33
**OV**	48.03	1.53	31.39	153.97	0.21	733.19	4.39	1.18	3.72	18.44	2.29	8.05	17.83	0.92	19.38	63.36	57.07	1.11	0.03	0.01	3.00
**PAAD**	1.09	0.74	1.47	118.77	11.55	10.28	64.37	1.35	47.68	35.75	19.77	1.81	11.8	14.88	0.79	96.76	9.75	9.92	0.03	0.07	0.43
**PRAD**	1.12	12.32	0.09	58.18	16.76	3.47	17.97	13.82	1.30	3.87	10.52	0.37	11.63	9.32	1.25	48.75	62.03	0.79	0.1	0.09	1.11
**READ**	0.34	23.63	0.01	128.55	1.48	86.86	36.94	9.87	3.74	6.19	3.64	1.70	18.15	2.76	6.58	51.59	100.65	0.51	0.89	0.04	22.25
**SARC**	0.41	162.87	0.00	0.1	24.69	0.00	7.03	9.47	0.74	2.11	21.43	0.10	2.73	6.08	0.45	100.17	93.5	1.07	0.04	0	
**SKCM**	10.68	10.33	1.03	0.14	78.63	0.00	2.52	33.24	0.08	1.78	78	0.02	2.81	7.88	0.36	45.57	35.47	1.28	0.04	0.04	1.00
**STAD**	0.93	28.22	0.03	135.23	32.08	4.22	31.52	3.33	9.47	28.52	39.28	0.73	15.93	8.77	1.82	136.48	38.22	3.57	0.1	0.05	2.00
**THYM**	368.97	5.26	70.15	5.16	0.05	103.20	1.88	0.07	26.86	2.63	0.2	13.15	12.19	0.62	19.66	18.24	56.25	0.32	0.02	0	
**UCEC**	13.31	2.91	4.57	63.12	0.18	350.67	7.14	7.18	0.99	33.66	6.15	5.47	11.76	4.8	2.45	31.83	179.2	0.18	0.05	0.05	1.00

BLCA, bladder urothelial carcinoma; BRCA, breast invasive carcinoma; CESC, cervical squamous cell carcinoma; COAD, colon adenocarcinoma; ESCA, esophageal squamous cell carcinoma; HNSC, head and neck squamous cell carcinoma; KICH, kidney chromophobe carcinoma; KIRC, kidney renal clear cell carcinoma; KIRP, kidney renal papillary cell carcinoma; LICH, liver hepatocellular carcinoma; LUAD, lung adenocarcinoma; LUSC, lung squamous cell carcinoma; OV, ovarian serous cystadenocarcinoma; PAAD, pancreatic adenocarcinoma; PRAD, prostate adenocarcinoma, READ, rectum adenocarcinoma; SARC, sarcoma; SCKM, skin cutaneous melanoma; STAD, stomach adenocarcinoma; THYM, thymoma; UCEC, uterine corpus endometrial carcinoma.
